# The Effect of UV-B Radiation on *Bufo arenarum* Embryos Survival and Superoxide Dismutase Activity

**DOI:** 10.3390/ijerph2006030006

**Published:** 2006-03-31

**Authors:** J. Herkovits, J. L. D’Eramo, O. Fridman

**Affiliations:** 1 Programa de Seguridad Química, Instituto de Ciencias Ambientales y Salud, Fundación PROSAMA, Paysandú 752 (1405), Buenos Aires, Argentina. Member of Consejo Nacional de Investigaciones Científicas y Técnicas (CONICET) de la Argentina.

**Keywords:** UV-B, Amphibian embryos, Superoxide dismutase

## Abstract

The exposure of *Bufo arenarum* embryos to 300–310 nm UV-B at a dose of 4,104 Joule/m^2^ resulted in 100% lethality within 24 hr while 820 Joule/m^2^ was the NOEC value for short-term chronic (10 days) exposure. The dose response curves show that lethal effects are proportional with the dose and achieve its highest value within 48 hr post exposure. The superoxide dismutase (SOD) activity in amphibian embryos for sublethal UV-B exposures was evaluated by means of UV-B treatments with 273 (A), 820(B), 1368(C) and 1915(D) Joule/m^2^ at 2 and 5 hours post irradiation. The SOD activity in units/mg protein in A, B, C and D at 2 hr after treatments were 80.72 ± 14.29, 74.5 ± 13.19, 39.5 ± 6.99 and 10.7 ± 1.89 respectively while for control embryos it was 10.88 ± 1.31. At 5 hr after treatments the SOD values were similar to those found in control embryos. The results confirm the high susceptibility of amphibian embryos to UV-B and point out that the SOD activity is enhanced by low doses of UV-B irradiation achieving significantly higher values than in control embryos at 2 hr post exposure.

## Introduction

Exposure to UV-B may result from both natural and artificial sources. The sun is the principal source of exposure for most people and wildlife. Decreases in stratospheric ozone levels from anthropogenic inputs of chlorinated fluorocarbons have resulted in an increased amount of harmful ultraviolet-B (UV-B, 280–315 nm) radiation, having serious consequences for living organisms. A 10% reduction in ozone could lead up to 15–20% increase in UV exposure [1]. The primary products generated by UV absorption are reactive species in a metastable excited state of free radicals such as superoxide. Dark chemical reactions then occur often within microseconds but they may last for hours, as in the case of the lipid peroxidation chain reaction. The ion superoxide induce peroxidation of fatty acids, affecting membrane lipids [2], provoke monobasic damage, strand breaks and reading frame shifting on DNA [3], e.g. denaturalization and/or degradation of proteins [4], and depolarization of polysaccharides [5]. The cellular consequences of damage due to UV radiation include changes in membrane permeability and membrane transport systems, activation of genes, mutation, inhibition of cell division, activation of viruses, etc., resulting in cellular death [6]. At high dose UV-B radiation enhance lipid peroxidation, a widely accepted general mechanism for cellular injury and death [7–9]. Photoreactivating enzymes activities (photolyase) are a common mechanism of protection against UV-B exposure [8, 9]; in some organisms, photoreactivation is the most important mechanism for the repair of cyclobutane pyrimidine dimers (CBPDs) [10], which are major cytogenic and mutagenic photoproducts in the DNA.

One of the major classes of antioxidant enzymes characterized in eukaryotic cells is superoxide dismutase (SOD), a family of metalloenzymes which catalyzes the spontaneous dismutation of superoxide anion to hydrogen peroxide and molecular oxygen. SOD is widely distributed in aerobic organisms and plays an important role in the control of radical superoxide levels in the cellular compartments [10]. Most oxidative enzymes are present in the skin of vertebrates [6], and under low dose of UV-B radiation, cells can generate an adaptive response through the induction of these stress proteins. These enzymes represent the first line of defense of aerobic cells against the toxic effects of free radicals. It is noteworthy that in the case of high UV-B irradiation, which causes lethality within few hours after treatment, no changes in the SOD, was registered in *Bufo arenarum* embryos [11].

The populations of many amphibian species, in widely scattered habitats, appear to be in severe decline. There is no known single cause for the declines, but their widespread distribution suggests the involvement of global agents such as increased UV-B radiation [12, 13]. Amphibians seems to be very The most susceptible to UV-B radiation at early life stages are the eggs and young forms producing that can suffer severe developmental abnormalities and effects on metamorphosis [13–15]. On the other hand the high susceptibility of amphibian embryos to various environmental pollutants [16–18] and their synergistic effects with UV-B radiation as in the case of Ni [19] could contribute significantly to the malformations and the worldwide decrease in amphibian population. In previous studies conducted with amphibian embryos the toxicity of various substances were reported as Toxicity Profile (TOP) curves by plotting concentration-response data for LC(EC)_90_, LC_50_ and LC_10_ for acute, short term chronic or even chronic exposure period. By this means isotoxicity curves were reported [20, 21]. The main purpose of this study is to report the dose response curves for UV-B exposure in *Bufo arenarum* embryos, the LC_10_, LC_50_ and LC_90_ plotted as TOP curves up to 10 days post irradiation (that is chronic effects in the case of early life stages), and the SOD activity in control and UV-B treated embryos. The possibility to employ amphibian embryos for the evaluation of the adverse effects of both natural and artificial sources of UV will be discussed.

## Materials and Methods

Adult *Bufo arenarum* captured in the surroundings of Buenos Aires City were purchased from a commercial suplice. The animals weighed 200–250 g and were housed in a dry tank at 18°C. Ovulation of females *Bufo arenarum* was induced by means of i.p. injection of homologous hypophysis. Oocytes were fertilized *in vitro* with a sperm suspension prepared in AMPHITOX solution (AS) [22]. Triplicate groups of 10 individuals obtained from three couples of parents were maintained in AS until the complete operculum stage, (Stage 25) [23].

Irradiation was performed by placing the uncovered Petri dishes containing the embryos under an UV-B light (300–310nm) with 4,56 watt/m^2^ of irradiantia (UVP M-20, UVP Inc Upland CA). Dose-response curves were evaluated by expressing triplicate groups of 10 embryos in 10 ml of AS to 820, 1368, 1915, 2460, 1730, 2730 3550, and 4100 Joule/m^2^ and maintaining them in 40 ml of AS during 10 days post irradiation. The duration of the UV-B exposures ranged from 3 to 15 min. for the lowest and highest doses, respectively. Triplicate groups of 10 individuals maintained in AS under standard (non-UV-B) laboratory lighting were used as controls. Embryo survival was evaluated daily up to 10 days post exposure. Dead embryos were removed. Mortality of the experimental embryos was statistically analyzed by means of PROBIT reporting LE_10_, LE_50_, and LE_90_ from 24 to 240 hr and was plotted as TOPs (isotoxicity) curves.

SOD activity was measured in duplicate groups of 20 embryos treated with irradiations ranging between 273 and 1915 Joule/m^2^, which represent a range of irradiations causing no to little lethality. Embryos at 2 and 5 hr post irradiation from each experimental group were kept in liquid nitrogen. For the determination of SOD activity, embryos were thawed and homogenized in potassium phosphate buffer 10 mM pH 7.0 with 1 mM EDTA. Homogenates were centrifuged 10 min at 10.000 rpm and SOD activity was determined in the supernatant. Assay was performed in triethanolamine-diethanolamine buffer (75 mM, pH 7.4), containing 3.0 mM EDTA; 1,5 mM MnCl_2_, 0,1 mM NADH, and 1,0 mM β-mercaptoethanol. Under these conditions O_2_^−^ is generated at a constant rate oxidizing the NADH. SOD inhibits this oxidation. An inhibition curve was obtained using various concentrations of supernatant. SOD activity was calculated as an I_50_ value, 1 unit being defined as the amount of enzyme that inhibits the oxidation of NADH by 50%. Enzymatic activities were expressed as units/mg of proteins measured in the supernatant by Lowry s method [24]. All SOD values are means+/−SE. One way ANOVA followed by Tukey′s test was performed with STATISTIX software. P values less than 0.05 were considered as significant.

## Results

### UV-B Toxicity and TOP Curves

**Figure 1:**
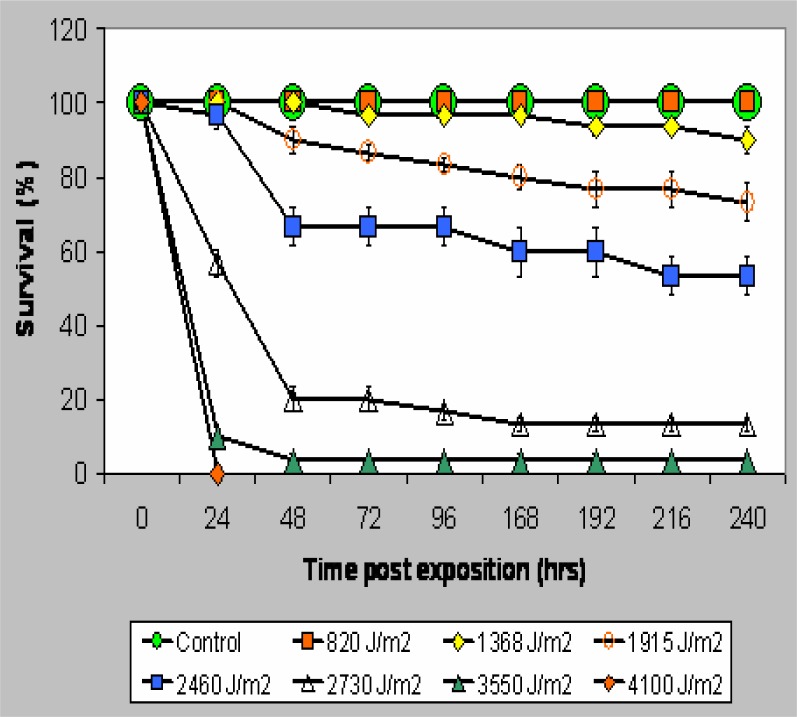
Dose response curves of UV-B radiation on *Bufo arenarum* embryos

Fig. 1 shows the dose response curves relating UVB exposure to lethality. 820 Joule/m^2^, 300–310 nm UVB radiation did not cause lethality in *Bufo arenarum* embryos up to 10 days post irradiation, whereas 2480 Joule/m^2^ was the maximal dose at which no lethality occurred within the initial 24 hours post exposure. All treatments over 1368 Joule/m^2^ resulted in a progressive increase in lethality up to 4100 Joule/m^2^ which caused 100% of lethality within 24 hr.

**Figure 2:**
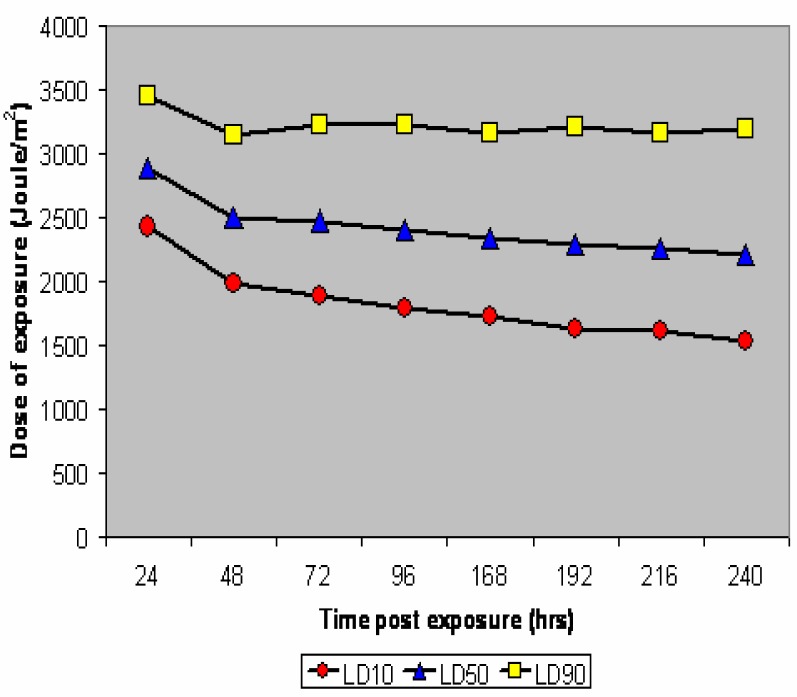
Top curves for UV-B in *Bufo arenarum* embryos

LE_10_, LE_50_, and LE_90_ values associated to the toxicity of UV-B on *Bufo arenarum* embryos from 24 to 240 hr post irradiation are plotted in Fig. 2. The cumulative mortality post UV-B irradiation resulted in a reduction of the dose required to cause a given adverse effect as the time post irradiation is expanded. The LE_50_ values for 300–310 nm of wavelength decreased from 2891 to 2209 Joule/m^2^ of exposure. A similar pattern is observed for LE_10_ (from 2424 to 1529 Joule/m^2^) and LE_90_ (from 3445 to 3191 Joule/m^2^).

Table 1 shows that the intervals of confidence of LE_50_ do not overlap with those of LE_10_ and LE_90_. The NOEC value was approx. 820 Joule/m^2^, while the LE_100_/24 hr post irradiation was approximately 4100 Joule/m^2^.

**Figure 3:**
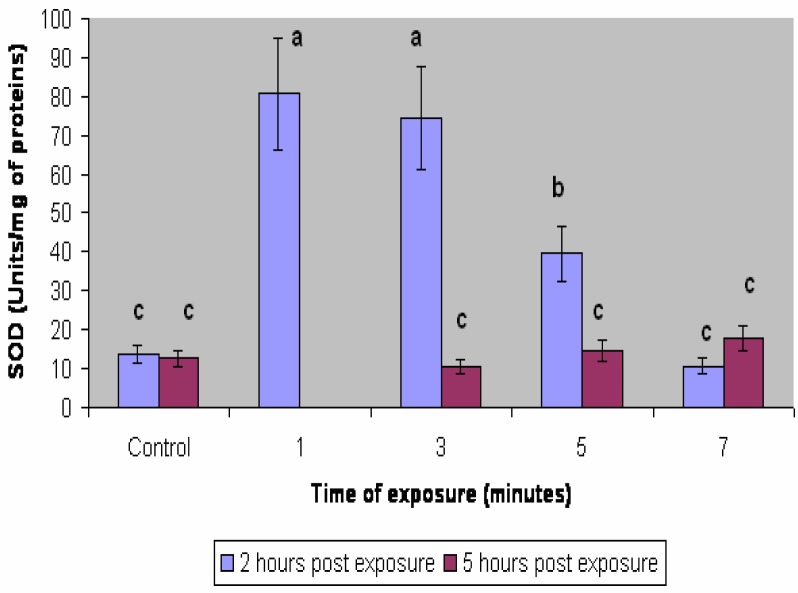
Effect of UV-B radiation on superoxide dismutase activity in *Bufo arenarum* embryos

SOD activities determined in embryos treated with low UV-B levels are shown in Fig 3. The maximal SOD response was achieved between 273 and 820 Joule/m^2^ 2 hr after irradiation. Slightly higher doses such as 1368 Joule/m^2^ resulted in a minor response in SOD activity but still significantly higher than in control embryos. Higher doses (e.g. 1915 Joule/m^2^) resulted in similar SOD activity as in control embryos. Five hours after exposure, SOD activity did not exhibit any significant differences with respect to control embryos.

## Discussion

As a general pattern, the dose-response and isotoxicity - TOP curves - [20] of UV-B treated *Bufo arenarum* embryos point out a proportional increase in mortality as the irradiation is increased with a cumulative lethal effect observed mainly within the initial 48 hr post exposure. In fact, the lethality observed in UV-B treated *Bufo arenarum* embryos increased only 1.31 times from 24 to 240 hr post exposure. Therefore, in the case of doses of UV-B irradiation exerting acute lethality, the initial 48 hr post exposure is the most critical period. This pattern of toxicity is similar to the adverse effects observed with some metals such as Al [20] and to some extent Cu [21] and Cd [25] in which it is not unusual to obtain around the same LC value during the initial 24 hr and up to 168 hr (short-term chronic) post exposure. Although in the case of metal toxicity the exposure condition is usually continuous, it is notwithstanding that based on uptake/tissue residue studies in amphibian embryos the plateau level is achieved for early developmental stages within one hr. after exposure [16].

Taking into account that in this study 1368 Joule/m^2^ of UV-B exerted only minimal lethal effects even after 10 days post exposure, it can be assumed that natural exposure to UV-B, that is around 1250 Joule/m^2^, have no lethal effects on *Bufo arenarum* embryos. The susceptibility of amphibian embryos and larvae to UV-B seems to vary significantly in different species. In fact, although the survival results for *Bufo arenarum* are in agreement with the data reported for Rana *arvalis*, Rana *temporaria* and *Bufo.bufo* [26], in the case of other anuran species such as, *Rana pipiens*, *Rana clamitans* and *Rana septentrionalis*, ambient levels of solar radiation were found to be lethal to all three species under exposure conditions that eliminated shade and refuge [13].

Amphibians have defenses against UV-B irradiation that can limit damage or repair it after exposure to this physical agent [27]. These include behavioral, physiological, and molecular defenses. These defenses differ interspecifically, with some species more able to cope with exposure to UV-B than others. The fact that *Bufo arenarum* pertains to the more resistant amphibian species to UV-B irradiation could be related to their dark pigmentation, a factor which could allow them to cope better than others in front of UV-B exposure. On the other hand, the highly significant increase in SOD activity in a wide range of sublethal irradiation conditions seems to be directly related to defense mechanisms against the oxidative stress exerted by UVB irradiation. It is noteworthy that in *Bufo arenarum* embryos and larvae SOD activity at 2 hr post exposure increases about 8 times in a wide range of doses from 200 to 800 Joule/m^2^; while at higher levels which could already exert lethality in the most UV-B sensitive embryos, SOD activity only increased about 2 times compared with controls. Exposures exerting higher letal effects resulted in no increase in SOD activity at least during the time period evaluated. It is noteworthy that at 5 hr post exposure in all experimental conditions SOD activity was found to recover to a similar level than in control embryos. These results point out that SOD activity could be related to active defense mechanisms against oxydative stress exerted by UV-B irradiation detectable in the case of sublethal exposure conditions. The high increase in SOD activity at 2 hr post sublethal UV-B exposure could be used as a biomarker of UV-B irradiation in amphibian embryos. SOD activity as a result of acute or chronic UV irradiation seems to differ very markedly in different biological models. In human blood mononuclear cells was also found an enhancement of SOD activity after UV-B radiation to 270 Joule/m^2^ but the increase was limited to 1.3-1.5-fold after a 3 h dark incubation period [28]. However in most studies like in C57 BL6 mice skin exposed to acute or chronic UV-B irradiation, SOD activity decreased sharply reaching a minimum at 18 hr after acute irradiation. [29]. In murine epidermis and the dermis after exposure to UV radiation (25 Joule/cm^2^, UV-A+UV-B), superoxide dismutase activities reached a minimum at 3 h postexposure with a recovery in the epidermis by 12 h and in the dermis by 120 h. [30]. It has been reported that ultraviolet radiation by generating reactive oxygen intermediates (ROIs) induces apoptosis. The acute lethality exerted by UV-B in amphibian embryos could be related to the apoptosis of a significant number of cells after high UV-B irradiation.

Due to the depletion of the stratospheric ozone, living organisms including humans are exposed to higher intensities of UV-B. On the other hand most artificial sources of UV, except for lasers, emit a spectral continuum of UV characteristic peaks, troughs and lines. These sources include various lamps used in medicine, industry, commerce, research and home. UV-induced biological effects depend on the wavelengths of the radiation. By summing the biologically effective irradiance over the exposure period, the biologically effective radiant exposure (Joule/m^2^ effective) can be calculated. The most used quantity for describing the erythemal potential of an exposure to UV-B is the number of erythemal doses represented by the exposure of UV-B that produces an erythema on a previously unexposed skin. It correspond to a radiant exposure at the maximum spectral efficacy for erythema (around 300 nm), of approximately 150 to 2000 Joule/m^2^ effective, about the same experimental conditions as conducted in this study. As 200 – 300 Joule/m^2^ effective is used as the value of 1 MED for comparative safety purpose for white skin, the fact that in *Bufo arenarum* embryos SOD activity at 2 hr post exposure increases about 8 times in a wide range of doses from 200 to 800 J/m^2^ could open the possibility to employ SOD activity, at least in this amphibian embryo, as an alternative test for erythema studies.

## Figures and Tables

**Table 1:** Lethal Exposition Toxicity and Intervals of Confidence of UV-B in *Bufo arenarum* embryos (Joule/m^2^)

*Time post exposition(hr)*	*LE_10_*	*LE_50_*	*LE_90_*
24	2424 (2244 – 2553)	2891 (2768 – 3030)	3462 (3249 – 3778)
48	1979 (1761 – 2124)	2512 (2359 – 2621)	3134 (2935 – 3478)
72	1892 (1384 – 2151)	2471 (2184 – 2749)	3227 (2875 – 4210)
96	1783 (1559 – 1946)	2397 (2250 – 2544)	3224 (2989 – 3609)
168	1728 (1504 – 1889)	2334 (2184 – 2479)	3153 (2924 – 3230)
192	1630 (1400 – 1796)	2285 (2127 – 2438)	3205 (2954 – 3615)
216	1611 (1384 – 1777)	2255 (2097 – 2405)	3156 (2910 – 3571)
240	529 (1299 – 1701)	2206 (2045 – 2364)	3191 (2927 – 3617)
